# Potato soup: analysis of cultivated potato gene bank populations reveals high diversity and little structure

**DOI:** 10.3389/fpls.2024.1429279

**Published:** 2024-07-18

**Authors:** Heather K. Tuttle, Alfonso H. Del Rio, John B. Bamberg, Laura M. Shannon

**Affiliations:** ^1^ Department of Horticultural Science, University of Minnesota, St. Paul, MN, United States; ^2^ U.S. Department of Agriculture (USDA)/Agricultural Research Service, Potato Genebank, Sturgeon Bay, WI, United States

**Keywords:** core subsets, autopolyploidy, ploidy detection, admixture, heterozygosity, multiploidy populations

## Abstract

Cultivated potatoes are incredibly diverse, ranging from diploid to pentaploid and encompass four different species. They are adapted to disparate environments and conditions and carry unique alleles for resistance to pests and pathogens. Describing how diversity is partitioned within and among these populations is essential to understanding the potato genome and effectively utilizing landraces in breeding. This task is complicated by the difficulty of making comparisons across cytotypes and extensive admixture within section petota. We genotyped 730 accessions from the US Potato genebank including wild diploids and cultivated diploids and tetraploids using Genotype-by-sequencing. This data set allowed us to interrogate population structure and diversity as well as generate core subsets which will support breeders in efficiently screening genebank material for biotic and abiotic stress resistance alleles. We found that even controlling for ploidy, tetraploid material exhibited higher observed and expected heterozygosity than diploid accessions. In particular group *chilotanum* material was the most heterozygous and the only taxa not to exhibit any inbreeding. This may in part be because group *chilotanum* has a history of introgression not just from wild species, but landraces as well. All group *chilotanum*, exhibits introgression from group *andigenum* except clones from Southern South America near its origin, where the two groups are not highly differentiated. Moving north, we do not observe evidence for the same level of admixture back into group *andigenum*. This suggests that extensive history of admixture is a particular characteristic of *chilotanum*.

## 1 Introduction

Potato is the third most important global food crop (FAO, 2019), because it is widely adapted and has a high nutrient to price ratio (Drewnowski and Rehm, 2013). It is unequivocally one of the most complete foods, containing significant dietary fiber, protein, vitamin C, B6, potassium, magnesium, iron and phytonutrients (King and Slavin, 2013; McGill et al., 2013; Brown et al., 1993; Liu, 2013; Beals, 2019; Navarre et al., 2019). Furthermore, potato grows well in harsh environments on six continents. It produces more calories and protein per unit land or water than any other staple crop (Renault and Wallender, 2000). Both India and China have undertaken national efforts to increase potato production as a way to support growing populations (FAO, 2019). As global populations expand and the climate changes, our reliance on potatoes will increase and breeders will need to develop varieties that can adapt to new environments.

Despite the agricultural and nutritional importance of potato, adoption of new potato varieties is slow and the most commonly grown potato variety in the US was developed over a century ago (Douches et al., 1996). Breeders need tools to facilitate the development of new varieties with increased biotic and abiotic stress resistance. The most effective tool for introducing new resistance alleles into potato is introgression from landraces and crop wild relatives (Jansky et al., 2013).

The USDA potato genebank in Sturgeon Bay WI is the most accessible repository of this germplasm for US breeders. Screening the genebank collection has identified new resistance alleles for pests and pathogens such as late blight (Karki et al., 2021), zebra chip (Mora et al., 2022), and Colorado potato beetle (Jansky et al., 2009). However, the genebank contains almost 2,000 accessions of cultivated potato and screening all of them is prohibitively difficult. Therefore, the first step to identifying valuable alleles within the collection is assembling the right screening panel. Building core collections within the genebank facilitates this process.

Core collections are an essential tool for empowering efficient screening of genebank material for use in breeding (Haupt and Schmid, 2020; Sokolkova et al., 2020; Phogat et al., 2021; Mufumbo et al., 2023; Santos et al., 2023; Shigita et al., 2023). The goal is to maximize the number of alleles evaluated while minimizing the number of individuals that must be screened. Such collections have been developed for a wide variety of crops using both morphological (Phogat et al., 2021; Santos et al., 2023) and genetic (Haupt and Schmid, 2020; Mufumbo et al., 2023; Shigita et al., 2023) data. While core subsets based on morphology and traits emphasized by breeders have advantages for use in prediction (Corak et al., 2019), they rely on the curators to foresee the total set of potentially relevant traits. New pathogens are continually arising (Duellman et al., 2020; Willbur et al., 2023) and mutating (Tran et al., 2022). Core subsets based on neutral markers maximize total diversity represented, even if the functionality of that diversity is not immediately obvious. Furthermore, genotyping gene bank collections with neutral markers provides insight into diversity and relatedness of the collected species.

The US potato genebank houses four core collections of wild potato species, *S. jamesii* (Bamberg et al., 2016), *S. fendleri* (Bamberg et al., 2016), *S. microdontum* (Bamberg and del Rio, 2014), and *S. demissum* (del Rio and Bamberg, 2020), and one core collection of diploid cultivated *S. chilotanum group phureja* (del Rio and Bamberg, 2021). Each of these were developed using AFLPs and validated with phenotyping data. However, individual taxa only represent a fraction of potato as a whole. Section petota includes four cultivated species and 107 wild relatives, ranging from diploid to hexaploid (Spooner et al., 2007, 2014).

Understanding how diversity is partitioned within and among these cultivated taxa facilitates their use in breeding. The history of potato, is complex. Cultivated potatoes were domesticated at least once from *S. candolleanum* (2n=2x=24) in the Andes near Southern Peru and Northern Bolivia more than 10,000 years ago (Camire et al, 2009). Potatoes were later cultivated in the highland equatorial conditions of Colombia and Venezuela as well as long day conditions in Chile and southern Argentina. Autopolyploidization of early landrace species, *S. tuberosum* groups *stenotomum* and *phureja* gave rise to *S. tuberosum* group *andigenum* (2n=4x=48) (Grun, 1990; Hawkes, 1990; Sukhotu and Hosaka, 2006). Later migration to coastal Chile led to the long-day adapted *S. tuberosum* group *chilotanum* (2n=4x=48) which is purported to have contributed most of the genetic background of *S. tuberosum* cultivars outside South America (King and Slavin, 2013). Understanding how diversity is partitioned within and among these taxa is essential to understanding potato’s history and guiding its future.

By all estimates, potato is highly heterozygous (Hardigan et al., 2017; Hoopes et al., 2022). However, descriptions of comparative levels of diversity between populations differ, in part because disparate ploidy levels complicate comparison. While tetraploid cultivated potatoes have higher heterozygosity and nucleotide diversity than diploid wild potatoes by some calculations (Hardigan et al., 2017), that relationship is reversed if SNPs are called more stringently (Huang et al., 2018) or if diploid cultivated potatoes are considered instead of tetraploid ones (Li et al., 2018). Across species, polyploids are generally more heterozygous than diploids due to the increase in number of alleles per locus (Meirmans et al., 2018). Therefore, fair comparison across cytotype requires correction for ploidy. When corrections are not made, the diversity in wild and cultivated diploid potato is under estimated (Bamberg and del Rio, 2020).

Another factor which complicates our understanding of how diversity is partitioned in potato, is the porous boundaries within section petota. While membership in petota is stable, relationships between taxa within the section are not (Gagnon et al., 2022). Taxonomy within section petota is made difficult by morphological similarity, phenotypic plasticity, allele loss, a mixture of sexual and asexual reproduction, recent species divergence, polyploidy, introgression, and multiple hybrid origin (Spooner and van den Berg, 1992; Spooner, 2009; Ames and Spooner, 2010; Cai et al., 2012; Huang et al, 2019; Zhou et al., 2020). There is extensive evidence for both hybridization in wild species (Hawkes, 1969; Ugent, 1970; Hawkes, 1990; Spooner et al., 2007; Tang et al., 2022) and for wild species introgression into cultivated US and European potato continually since domestication (Hardigan et al., 2017; Hoopes et al., 2022; Meng et al., 2022). The genebank collection provides the opportunity to investigate the extent to which the landraces exhibit the same history of admixture as wild and US/European improved potatoes.

In this study, 730 accessions of diploid wild and diploid and tetraploid cultivated individuals were genotyped using a genotyping-by-sequencing (GBS) approach (Elshire et al., 2011). We interrogated this data set to address three questions. (1) How is diversity, both individual heterozygosity and population level allelic diversity, structured within cultivated potato? (2) Does the pattern of admixture observed in wild and commercial potato also describe the land race taxa? and (3) What is the ideal composition of core subsets for screening this portion of the genebank collection?

## 2 Materials and methods

### 2.1 Plant materials and sequencing

The US potato genebank in Sturgeon Bay, Wisconsin holds 1,445 accessions of cultivated potato. This assemblage represents years of collections in fields and markets some from planned collecting trips and some incidental, as well as contributions from researchers. We genotyped a subset of the collection, chosen with an eye to maximizing phenotypic diversity, containing 730 diploid, tetraploid and pentaploid accessions (**Supplementary Table S1**), using genotyping-by-sequencing (GBS) (Elshire et al., 2011) with the EcoT22 enzyme and phased adapters on the Illumina HiSeq platform at the University of Minnesota Genomics Center. According to passport data these 730 accessions consisted of 72 diploids including: *S. juzepczukii* (6), *S. berthaultii* (1), *S. brevicaule* (1), *S. ajanhuiri* (3), *S. phureja* (27), *S. stenotomum* (11), *S. boliviense* (20) and *S. tuberosum* group *chilotanum* (3). The 641 tetraploid accessions included: group *andigenum* (301), group *chilotanum* (333), *S. juzepczukii* (4), *S. phureja* (1) and *S. ajanhuiri* (2). Group *andigenum* accessions were primarily South American, coming from Peru, Bolivia, Colombia, Ecuador, Chile, Argentina, Uruguay, Venezuela and Brazil. Group *chilotanum* included accessions from North America, South America, Central America, Europe, Asia, Russia and Africa. Pentaploid accessions consisted of 14 *S. curtilobum* with two samples of group *chilotanum*. Triploids were not included in the genotyping panel. There was one accession for which the passport data did not include: taxa, clone name, or ploidy.

Some individuals, in particular those from group *chilotanum* in North America and Europe are named cultivars. These individuals are highly selected. The land race individuals including *S. juzepczukii*, *S. ajanhuiri*, *S. phureja*, *S. stenotomum*, and many of the *S. tuberousum* individuals from South America are random seedlings selected from genebank populations maintained as true seed. The wild individuals, most notably *S. boliviense*, are from a single wild population. Since we are only comparing genome wide patterns of diversity, rather than functional alleles, and we expect those to be similar in selected clones (ie cultivars) and individuals arising in crosses made from selections (ie the landrace populations), we can make comparisons across cultivated populations. However, since the *S. boliviense* is from a single population it is included only when an outgroup is needed for analysis. *S. boliviense* is removed from other analyses to avoid making unfair comparisons between representatives of a species as a whole and representatives of a single population (Bamberg and del Rio, 2020). Similarly, all taxa with only one representative were removed.

### 2.2 Sequence read alignment and variant calling

Reads were checked for quality with FASTQC 0.11.5 (Andrews, 2010) and adapters were removed with Cutadapt version 1.18 (Martin, 2011). Each sample was aligned to the Phureja DM v4.04 reference genome (Hardigan et al., 2016) with Bowtie 2 version 2.2.4 (Langmead and Salzberg, 2012) using Phred +33 encoding. We used Samtools version 1.9 (Danecek et al., 2021) to create bam files and report the number of mapped reads. Samples with less than 150,000 mapped reads were removed (**Supplementary Table S2**). Since we wanted to make comparisons of diversity within and among ploidy levels, we created three separate panels for our analyses; diploid, tetraploid and a third, combined panel (**Table 1**). SNP genotypes were called twice, once before ploidy correction and again after ploidy correction. All steps prior to ploidy correction were based on the combined panel. Once ploidy estimation by computational methods either confirmed or rejected the original ploidy calls, samples were removed or added to the panels and genotypes for single ploidy panels were called once again. BCFtools version 1.10.2 and samtools mpileup version (Danecek et al., 2021) were used to generate genotype likelihoods, call SNPs using the multiallelic caller and remove indels. Filtering thresholds appropriate for each panel were used (**Table 2**). VCFtools version 0.1.16 (Danecek et al., 2011) was used to remove unmapped reads, filter SNPs and extract biallelic SNPs at sites with no more than 40% missing data.

**Table 1:** Datasets used for analysis.

Dataset	Number of SNPs	Number of individuals	Application
Diploid, Naive	5148	56	All analyses
Tetraploid, Naive	4720	497(183)	Rho, Structure, PCoA, D statistics
Tetraploid, Simulated	4720	497	Heterozygosity, Gis
Combined, Naive	7810	553 (235)	Fst, Structure, PCoA, D statistics
Combined, simulated	7810	553	Heterozygosity, Gis

The diploid panel did not have sufficient individuals to create a dataset using simulated population structure. For analyses that strived to estimate population structure such as STRUCTURE, PCoA and Fst/Rho we used naive datasets which contained no more than 20 individuals from each population. Total number of individuals in these analyses are in parentheses.

**Table 2:** Filtering thresholds for each panel.

Filter	Diploid	Tetraploid	Combined
MQ	<20	<20	<20
GQ	<30	<60	<60
QUAL	<30	<40	<30
MAF	<0.02	<0.02	<0.02
DP	<5	<7	<5

Alignments with values below the threshold were removed. Tetraploids had better genotype quality and site quality over sequenced diploid individuals but did not have sufficient read depth to correctly discern genotypes. Sequencing error rate is 0.02 for all the data used.

Over 60X coverage is required to definitively distinguish between heterozygous classes in tetraploids (Uitdewilligen et al., 2015). Few markers in the tetraploid panel met this requirement. Therefore, we used PolyRAD (Clark et al., 2019) to re-call the most probable genotypes in panels containing tetraploid individuals. VCFs for the tetraploid and combined panel were read into the R (v4.2.1; R Core Team, 2021) environment using VCF2RADdata, only retaining variants that were called in at least 109 individuals (~20% of the population) and where the minor allele was in a minimum of 2 individuals. Variants in the diploid panel were only retained if they were present in at least 10 individuals (~20% of the population) with the minor allele present in at least 2 individuals. To filter loci in the combined panel, we separated individuals by ploidy and removed markers with a Hind/He less than 0.5 and greater than 0.75 for diploids and tetraploids, respectively. Genotypes were re-called by simulating population structure with individuals assigned to groups based on PCA. We created an additional dataset for each of the three panels that used a naïve model to call genotypes so as not to bias our population structure estimates for a total of six data sets (**Table 1**). After re-calling genotypes, loci with more than 10% missing data were removed and remaining missing data was filled in based on overall allele frequencies. For the combined panel and the tetraploid panel, dosage of polyploids was restored in Genodive version 3.0 (Meirmans, 2020) with resampled alleles.

### 2.3 Ploidy estimation

For multiple accessions in the data set, the passport data indicated a ploidy that conflicted with the known ploidy for the assigned taxa. The ploidy included in the passport data was determined using root tip squashes upon the addition of accessions to the genebank (Ordoñez et al., 2017). In order to confirm passport data, we estimated ploidy for each accession using two methods. First, histograms of allele frequencies across all markers were plotted for each individual to observe the number of peaks (Ellis et al., 2018). There are n+1 genotypic classes at ploidy n, therefore the ploidy level is equal to the number of histogram peaks minus one. We also ran GBS2ploidy with three settings (diploid vs. tetraploid, diploid vs. triploid vs. tetraploid, and diploid vs. triploid vs. tetraploid vs. pentaploid) (Gompert and Mock, 2017). Posterior estimates of allelic proportions were estimated with mcmc.nchain =2, mcmc.step = 10,000, and mcmc.burnin = 1000 and mcmc.thin = 2. After estimating allelic proportions, we performed ploidy estimations on the three datasets. We retained samples for which both methods of ploidy estimation confirmed the passport data.

### 2.4 Genetic diversity

With the goal of elucidating how diversity is distributed, we divided the panels into populations based on geography, species, and ploidy level. Countries were grouped together in an attempt to form reasonably sized populations. *S. phureja*, *S. boliviense*, and *S. stenotomum*, were each treated as a single taxa-based population. group *chilotanum* was further divided into geographic populations: Europe, US/Canada, Mexico/Guatemala, Brazil/Ecuador/Colombia, Peru, Bolivia/Argentina/Uruguay, and Chile. Similarly, group *andigenum* was subdivided by geography: Colombia/Venezuela, Ecuador, Peru, Bolivia, and Argentina/Chile. Location, reflects the best information we have based on collection site or breeder location, but may not reflect original geography of the clone. Species level diversity statistics such as expected heterozygosity (H_S_), observed heterozygosity (H_O_) and an inbreeding coefficient (G_IS_) that is analogous to F_IS_, were calculated in GenoDive (Meirmans, 2020) with the panels where genotypes were called taking population structure into account. All panels were bootstrapped ten times to obtain 95% confidence intervals.

### 2.5 Population structure

We quantified differentiation within and across ploidy, species, and geographic region using F_ST_ and rho (⍴) (Ronfort et al., 1998; Meirmans et al., 2018) calculated in GenoDive (Meirmans, 2020). We used ⍴ for the single ploidy panels and F_ST_ for the combined ploidy panel. Differentiation statistics are sensitive to sample size imbalance; therefore, we subsampled each population to retain no more than 20 individuals. Each population was resampled ten times and bootstrapped to determine 95% confidence intervals.

In order to visualize patterns of admixture between populations we used STRUCTURE (Pritchard et al., 2000). For all three panels, we used an admixture model that infers alpha with a 10,000 burn-in period and 10,000 MCMC replications. Lambda was set to 1.0. Priors used to parameterize the assumed probability models were set to default. This process was repeated for values of k between 1 and 6 for naive forms of the diploid, tetraploid and combined datasets. Because STRUCTURE does not handle multiple cytotypes, the naive combined panel was diploidized before analysis (Meirmans et al., 2018). We also used principal coordinate analysis (PCoA) within GenoDive (Meirmans, 2020). All results were plotted and visualized using the ggplot2 package version 3.4.1 (Wickham, 2011) in the R statistical environment version 4.2.1 (R Core Team, 2021).

Formal tests of admixture between individual populations were performed using Dsuite Dtrios (Malinsky et al., 2021) to calculate Patterson’s D (ABBA/BABA) statistics. Using all individuals in the naive combined panel, we first looked for possible admixture between each subpopulation of *S. tuberosum* group chilotanum and *S. tuberosum* group *andigenum* (tetraploid group *andigenum*, *S. phureja*, and *S. stenotomum*). We then examined admixture between each subpopulation of *S. tuberosum* group *andigenum* and *S. tuberosum* group *chilotanum*. *S. boliviense* was used as an outgroup and a jackknife block size of 150 was used to account for linkage. P-values were corrected for multiple comparisons using false discovery rate (FDR). Statistics were visualized in the R environment.

### 2.6 Core subset selection

In the interest of identifying core subsets, we used two software programs with different core collection algorithms. CoreHunter 3 (De Beukelaer et al., 2018) and GenoCore (Jeong et al., 2017) were used to generate core subsets for each panel using an allele coverage metric (CV) which maximizes the proportion of the total alleles observed in a subset. For each panel, multiple core sizes were created and evaluated for their ability to capture marker diversity.

## 3 Results

### 3.1 Alignment and ploidy estimation

After aligning all 730 individuals to the V4.04 phureja DM reference genome, 29 individuals were removed due to insufficient mapped reads (**Supplementary Table S2**). Examination of allelic ratios and heterozygosity confirmed the passport ploidy data of 613 of the 701 remaining accessions (**Supplementary Tables S3**, **S4**). Of these 613 accessions, there were 553 tetraploids including group *chilotanum* (303) and group *andigenum* (247). The final data set contained 54 diploid individuals including *S. stenotomum* (9), *S. boliviense* (20), and *S. phureja* (25) All putative pentaploid individuals were removed because we were unable to confirm the ploidy computationally.

### 3.2 Genotype calling and variant filtration

Genotypes were called separately for each panel, using methods and filtering thresholds applicable to the ploidy and panel size (**Table 2**). After initial filtering, we identified 7,296 SNPs in the diploid panel with an average of 26% missingness per site and 6.78x average read depth. Subsequent filtering in polyRAD and GenoDive left the diploid panel with 5,135 SNPs. The tetraploid panel contained 5,742 SNPs with an average of 26% missingness per site, 8.03x average site read depth and after additional filtering 4,720 SNPs were retained. The combined panel contained 9,668 SNPs after initial calling and filtering of genotypes with an average of 22% site missingness and 6.8x average read depth. Additional filtering left the combined panel with 7,851 SNPs. The Hind/He filter in PolyRad is intended to remove paralogs and none of the fixed heterozygous loci observed in other potato GBS panels (Bamberg et al., 2021) remained after filtering. A total of 1,264 SNPs were shared between diploid, tetraploid and combined datasets (**Supplementary Figure S1**).

### 3.3 Estimates of genetic diversity and inbreeding

**Figure 1:**
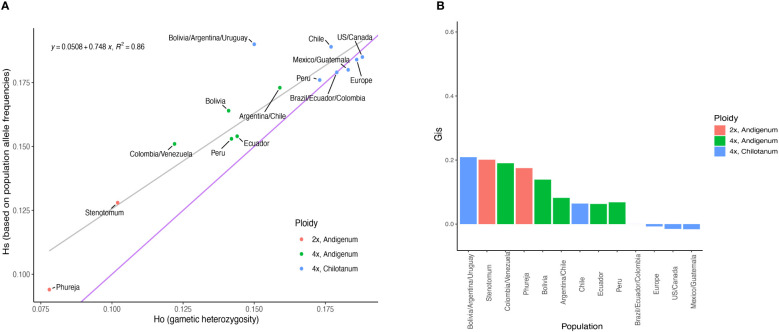
Diversity statistics from the combined panel. **(A)** Expected heterozygosity versus observed heterozygosity. The gray line is a fitted regression line with the equation given. The blue line indicates equal values for observed and expected heterozygosity. **(B)** Inbreeding coefficients for each population. Numeric values can be found in **Supplementary Table S3**.

By most measures, tetraploids had higher heterozygosity (H_O_ = 0.190, H_S_ = 0.239) than diploids (H_O_ = 0.112, H_S_ = 0.140) even when using methods that account for ploidy (**Figure 1A**, **Supplementary Table S3**). Group *chilotanum* was the most heterozygous, followed by tetraploid group *andigenum*, and the cultivated diploids. Using SNP panels derived from only a single ploidy all levels of heterozygosity were higher, as expected, but the relative levels of heterozygosity between populations were consistent. While tetraploid populations exhibited few private alleles (1 in Europe and 1 in Peruvian andigenum), all diploid populations did, with *S. bolivense* having 1,929 private alleles, more than four times the next highest population (**Supplementary Figure S2**).

All populations except Northern group *chilotanum* exhibit some degree of inbreeding (**Figure 1B**). With the exception of the populations from Bolivia, Argentina, and Uruguay (G_IS_ = 0.209), group *chilotanum* populations exhibited the lowest in breeding overall (G_IS_ = 0 – 0.064) and diploid populations the highest (G_IS_ = 0.175 – 0.201).

### 3.4 Population structure and admixture

**Figure 2:**
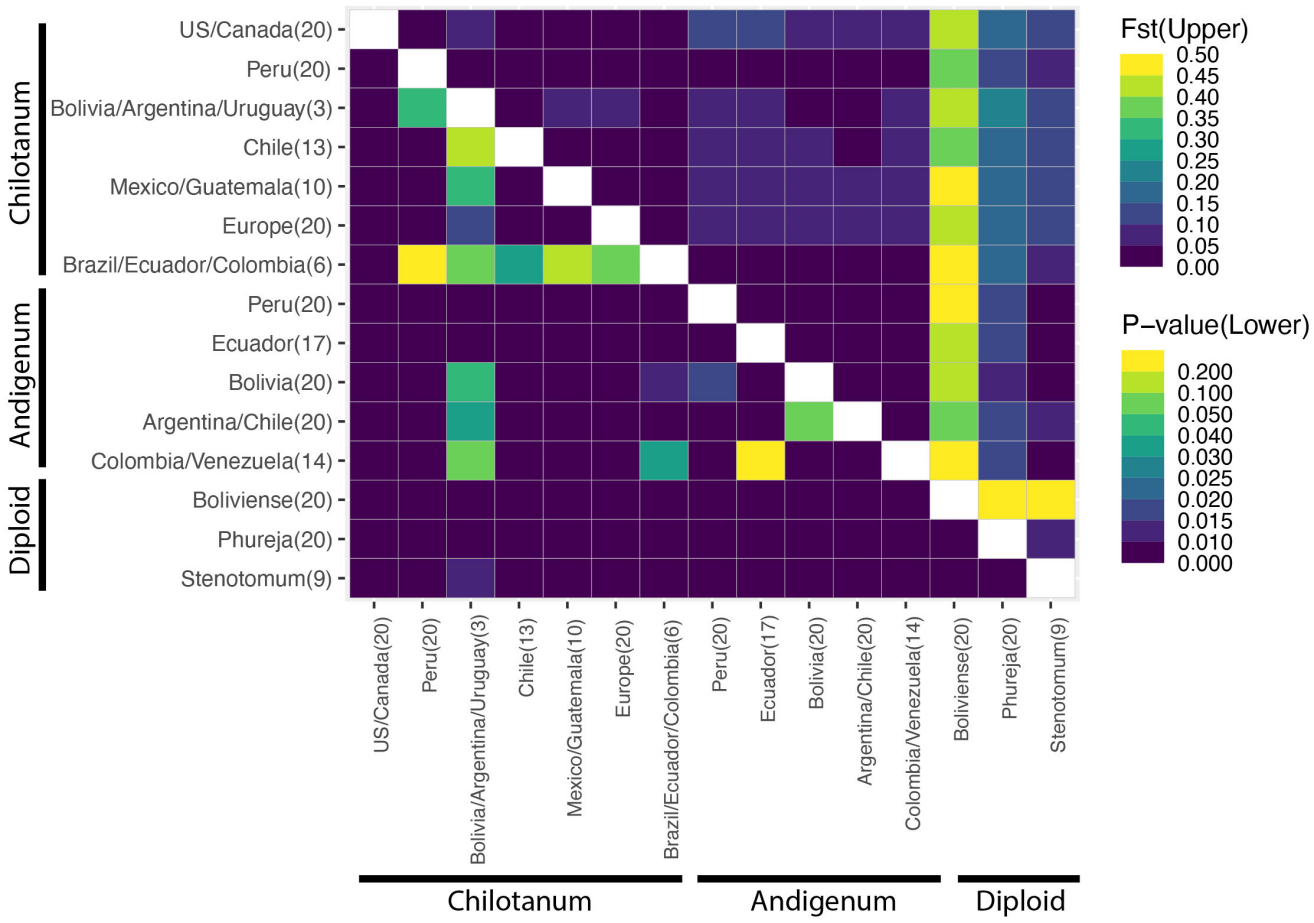
Fst comparisons for the combined panel (upper triangle). Fst is most appropriate for calculation of population structure across cytotype (Meirmans et al., 2018). The lower triangle indicates significance (p) values. Fst values can be found in **Supplementary Table S4**. Boliviense is the only wild species included in this analysis.

**Figure 3:**
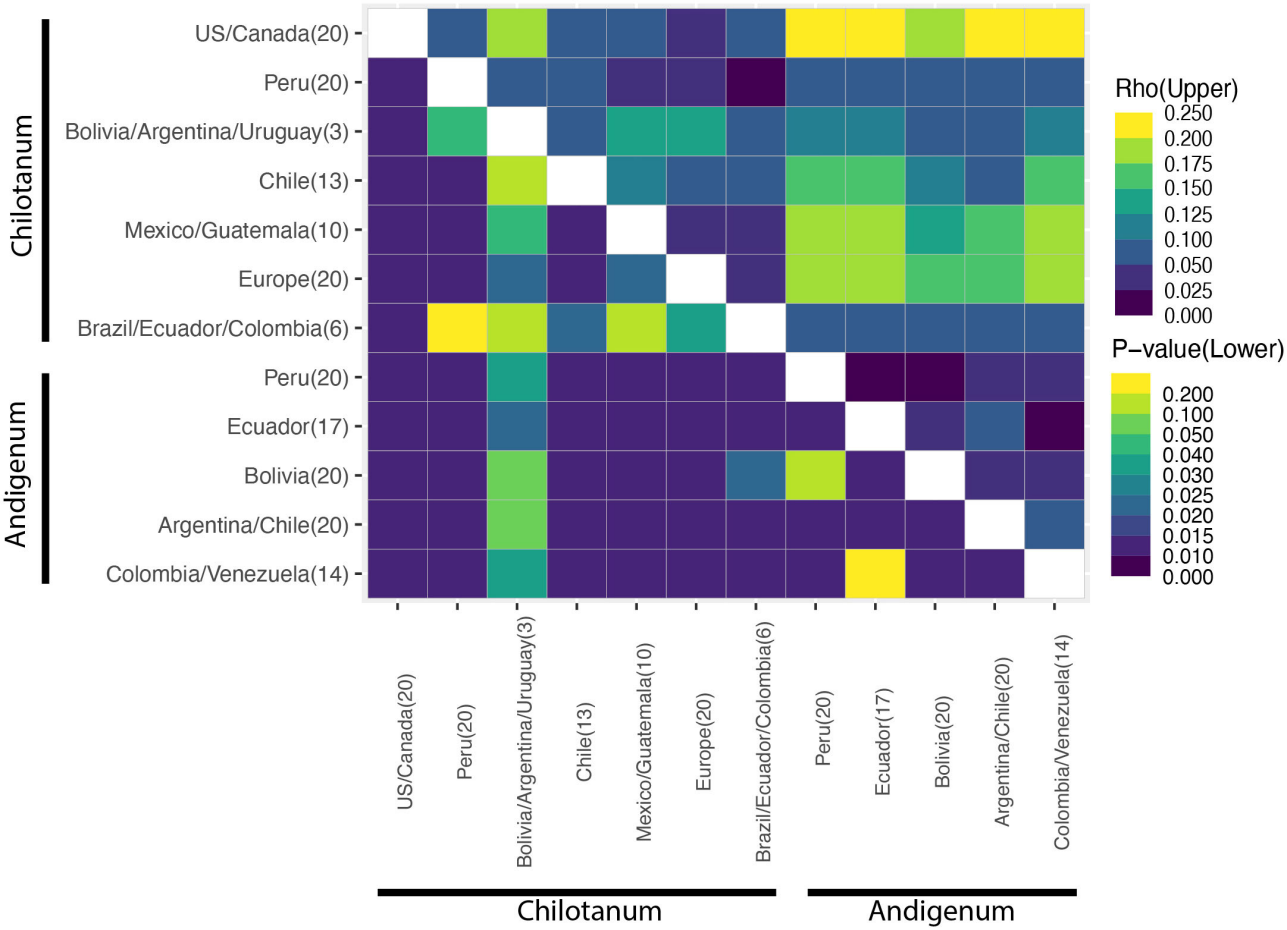
Rho (⍴) values for the tetraploid panel. ⍴ is most appropriate for calculation of population structure for autopolyploids (Meirmans et al., 2018). The lower triangle indicates significance (p) values. Analysis of the diploid panel can be found in **Supplementary Figure S3**. All ⍴ values can be found in **Supplementary Tables S5** and **S6**.

Based on F_ST_ there is little differentiation among tetraploids, with group *chilotanum* from the US and Canada and group *andigenum* from Peru exhibiting the most differentiation (F_ST_ = 0.109) (**Figure 2**, **Supplementary Table S5**). When using the tetraploid SNP panel to calculate ⍴ (rho), a more complicated pattern emerges (**Figure 3**, **Supplementary Table 6**). Northern group *chilotanum* (US, Canada, Mexico, Guatemala, and Europe) is distinct from group *andigenum* (⍴ = 0.14 – 0.236) and the group *chilotanum* material from Bolivia, Uruguay, and Argentina (⍴ = 0.144 – 0.181). Group *chilotanum* samples from Peru, Brazil, Ecuador, and Colombia were not differentiated from any of the tetraploid material. Subspecies pairs from similar geographic areas tended to have low ⍴-values, specifically the Bolivian pair (⍴ = 0.067), the Chilean pair (⍴ = 0.093), and the Colombian pair (⍴ = 0.066).

F_ST_ and ⍴ values are similar for the diploids (**Figure 2**, **Supplementary Figure S3**, **Supplementary Tables S5**, **S7**). In both cases *S. bolivense* is the most differentiated (⍴ = 0.762 – 0.786) and *S. stenotomum* and *S. phureja* are the most similar (⍴ = 0.117). *S. bolivense* is distinct from all tetraploids (F_ST_ = 0.386 – 0.483) and for the most part, so are *S. phureja* and *S. stenotomum* (F_ST_ = 0.574-0.607). *S. stenotomum* is highly similar to group *andigenum* (F_ST_ = 0.034 – 0.068) and group *chilotanum* from Peru, Brazil, Ecuador, and Colombia (F_ST_ = 0.066 – 0.074) and slightly less similar to other group *chilotanum* (F_ST_ = 0.104 – 0.125).

**Figure 4:**
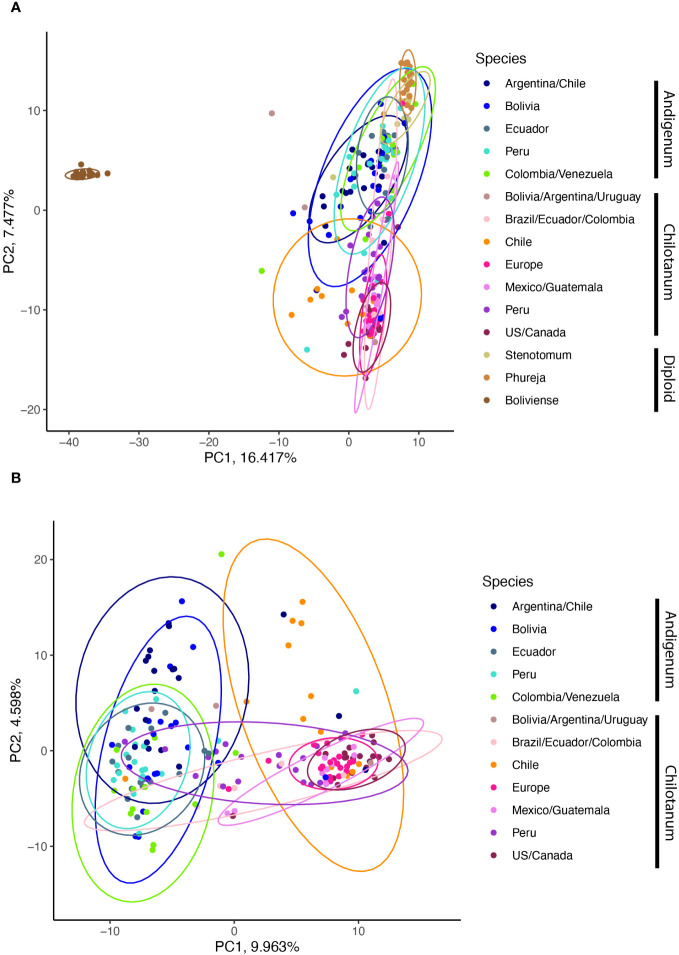
**(A)** Principle coordinate analysis from the combined panel. Boliviense, in brown is a wild species. **(B)** PCoA of the tetraploid panel.

PCoA distinguishes between the two subspecies with the first PC using just the tetraploid data (9.963% of the variation explained) (**Figure 4B**) and the second PC in the combined panel (7.477% of the variation explained) (**Figure 4A**). In both cases there are overlapping sets. The second PC in the tetraploid data extracts what is potentially a geographic component, with individuals from Chile and Argentina in both subspecies having the largest values.

**Figure 5:**
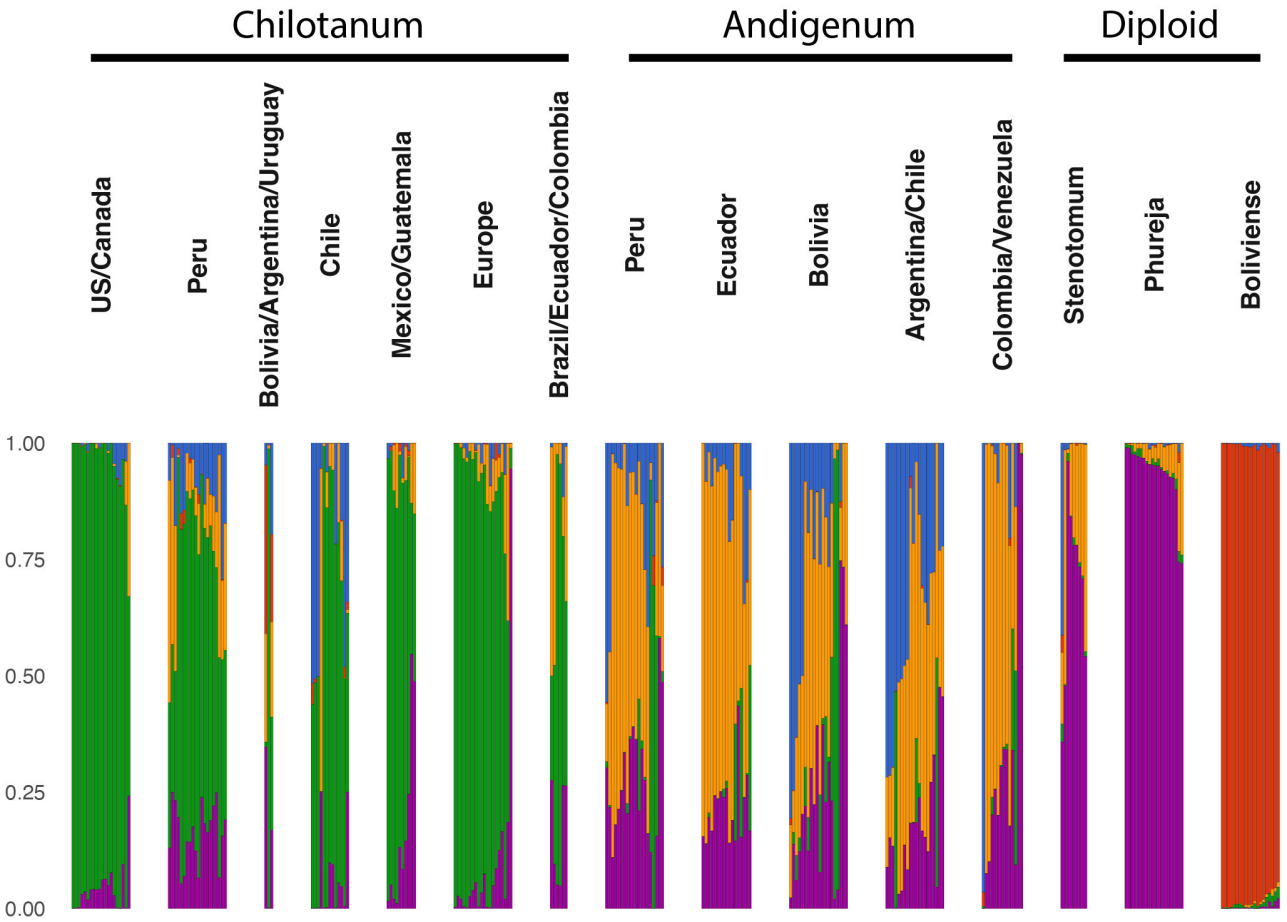
STRUCTURE (Pritchard et al., 2000) analysis for the combined panel using k=5. Large populations were subsampled to include 20 individuals. STRUCTURE analysis for the separately analyzed panels can be found in **Supplementary Figures S4** and **S5**. All groups are cultivated germplasm with the exception of Boliviense.

Analysis with STRUCTURE suggested five distinct ancestry components (**Figure 5**). The *S. bolivense* component (red) is found primarily in the mostly unadmixed *S. bolivense* population but also contributes to group *chilotanum* from Bolivia, Argentina and Uruguay. The cultivated diploid component (purple) is almost entirely unadmixed in *S. phureja* and dominant in *S. stenotomum*. It also appears in all the tetraploids with group *andigenum* and group *chilotanum* from Peru having the most diploid ancestry as compared to other tetraploids. This split between wild and cultivated diploids is replicated when the diploid panel is considered alone (**Supplementary Figure S4**). As expected, the tetraploid germplasm has a component for each subspecies; *chilotanum* in green and *andigenum* in yellow (**Figure 5**). There is some *chilotanum* presence in most group *andigenum* populations, with a notably large component in the Argentinian/Chilean population. Similarly, there is *andigenum* ancestry in all the group *chilotanum* populations with largest component in Peru and in all the diploid populations. There is a fifth (blue) component which appears most strongly in Chilean group *chilotanum* and Argentinian, Chilean, and Bolivian, group *andigenum*. This split between the two subspecies with a third potentially geographic component is replicated in the separate analysis of the tetraploid material (**Supplementary Figure S5**).

**Figure 6:**
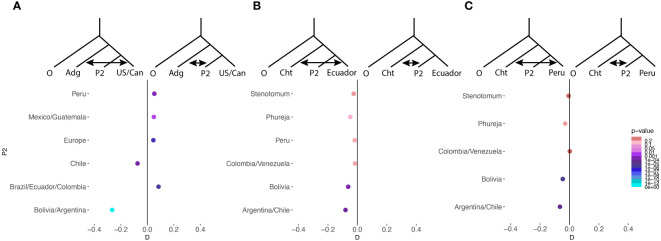
D statistics were used as a formal test of admixture between specific populations. In all cases *S. boliviense* was used as the outgroup. In **(A)** admixture between group *andigenum* as a whole and individual populations of *chilotanum* is tested. P2 is a different population in each test which is listed vertically along the side. A negative D statistic indicates admixture between *andigenum* as a whole and US/Canadian *chilotanum*, while a positive D statistic indicates admixture between P2 and *andigenum* as a whole. The magnitude of D reflects the amount of admixture, while the color of the point indicates the significance of the effect. In **(B, C)** admixture between *chilotanum* as a whole and individual populations of *andigenum* is tested and represented similarly to **(A)**.

In order to examine admixture between specific populations we used D statistics (**Figure 6**). We found admixture between the group *chilotanum* from the US and Canada, Europe, Peru, Mexico, Guatemala, Brazil, Ecuador and Columbia and other cultivated potato. We did not see admixture between non-group *chilotanum* cultivated potato and *chilotanum* from Chile, Bolivia, and Argentina (**Figure 6A**). When we examined group *chilotanum* geneflow with individual populations in group *andigenum*, we found some evidence of geneflow into Ecuador and Peru but none into most populations (**Figure 6B**). This suggests that for the majority of populations the direction of geneflow is from group *andigenum* into group *chilotanum*.

### 3.5 Core subsets

To determine how many individuals would be needed to capture most of the diversity in the genotyped collection, we created two sets of core subsets using Core Hunter 3 and GenoCore software, for the combined panel in this study. Both methods identified 329 individuals which included all alleles detected in the population (**Table 3**, **Supplementary Table S8**, **S9**). Although each of these subsets contained 65.3% of the total population, they differed by 44 individuals. Both were 3.3% diploid and slightly weighted toward group *andigenum* over group *chilotanum*, although CoreHunter produced a panel that had more *chilotanum* individuals than GenoCore. Further analyses showed that with 100 individuals, a coverage of 97% of the genetic diversity in this collection can be achieved, while 90% coverage is possible with just 50 individuals. With 20 individuals 70-77% of coverage is achieved. Recommended subsets are listed in **Supplementary Table S8**, **S9**.

**Table 3:** Statistics from the two software programs used to select core subsets.

Software	Number of individuals	Percent of individuals	Percent coverage	Percent chilotanum	Percent andigenum	Percent diploid	Percent tetraploid
GenoCore	10	1.80	61.81	40	40	20	80
	20	3.60	70.06	40	50	10	90
	30	5.41	83.47	33	57	10	90
	40	7.20	87.77	32.5	60	7.5	92.5
	50	9.01	90.62	36	56	8	92
	100	18.02	96.73	35	60	5	95
	329	59.28	100	41.7	55	3.3	96.7
CoreHunter3	10	1.80	63.78	50	50	0	1
	20	3.60	77.45	45	50	5	95
	30	5.41	84.42	33.3	63.3	33	96.7
	40	7.20	88.54	37.5	57.5	5	95
	50	9.01	91.26	34	62	4	96
	100	18.02	97.00	32	64	4	96
	329	59.28	100	42	54.7	3.3	96.7

## 4 Discussion

### 4.1 Comparison of the genotyping panels

We organized the individuals into three genotyping panels which were analysed separately. The first included all the diploids, the second contained the tetraploids, and the third was a combined panel. Although the combined panel contained all the individuals in the first and the second panel there were substantial differences in the SNPs detected in each panel (**Supplementary Figure S1**). Only 1,264 SNPs were found in all three panels. The combined panel contained 2,980 SNPs not identified in the other panels, an expected consequence of an increase in sample size. More surprisingly, the diploid panel contained 2,756 SNPs not present in other panels. This is likely due to alleles specific to diploid species. While only 5 private alleles, all in *S. bolivense*, were found in the combined panel, 1,929 were found in the diploid panel. When the diploids make up a smaller proportion of the population, diploid specific SNPs are less likely to meet even very low quality thresholds. This calls into question the validity of the SNPs uncovered only in the diploid panel, but also suggests that we are under counting SNPs unique to the diploids in the combined panel, which is likely to artificially lower heterozygosity measures. However, even when the ploidy specific panels are analysed *phureja* and *stenotomum* exhibit lower observed and expected heterozygosity than the tetraploids and show higher levels of inbreeding, suggesting that this is not an artefact of ascertainment bias.

Ascertainment bias has been implicated in over estimating tetraploid group *chilotanum* diversity, particularly referencing studies using the SolCAP array (Bamberg and del Rio, 2020). GBS reduces ascertainment bias through the use of denovo SNP discovery in the analysed population (Glaubitz et al., 2014). However, as seen in this study, differences in sample sizes can introduce bias in SNP calling, which in this case may be inflating tetraploid diversity. The choice of reference genome can also introduce bias (Glaubitz et al., 2014), although since the potato reference is a monoploid developed from diploid *phureja* this bias is likely to result in an underestimation of tetraploid diversity. Furthermore, GBS is inherently low coverage and relies on imputation particularly in polyploids (Clark et al., 2019). When missing data is not imputed and left missing it results in an underestimation of heterozygosity at loci with high missingness (Bamberg et al., 2021). However, when there is extensive structural variation, as seen in tetraploid potato (Pham et al., 2017; Hoopes et al., 2022), true missing alleles are often incorrectly imputed to common genotypes, thus underestimating tetraploid diversity (Della Coletta et al., 2021). Any and all of these conflicting sources of bias could affect the heterozygosity values we report.

It is important to note that, GBS data represents only a fraction of the genome, and only segregating sites were examined. Therefore, the heterozygosity values reported are only meaningful in comparison to each other rather than as absolute values. Heterozygosity in potato is a subject of debate with reports varying. Whole genome sequencing studies, which present the most complete least biased data, report about 1.5% heterozygosity in wild diploids (Aversano et al., 2015; Leisner et al., 2018; Hosaka et al., 2022), 1.73 – 4.48% in cultivated diploids (Kyriakidou et al., 2020), and 5 – 8% in cultivated tetraploids (Hoopes et al., 2022).

Furthermore, our panel is not the result of systematic sampling of global cultivated potato diversity, but rather a subset of a genebank collection. The genebank cultivated collection was built up over time through a variety of collection trips and donations. While the goal of the genebank collection is to preserve and make available the full range of potato genetic diversity, it is, of course, possible that sampling was somehow biased. In particular, our sample may not represent extant diversity as most accessions in genebanks were collected well over twenty years ago (Sotomayor et al., 2023). However, the agreement between our results and previous studies on other samplings of potato diversity, in particular the cultivated collection of the International Potato Center (CIP) in Lima Peru (Ellis et al., 2018), suggests the US genebank collection is in fact representative of cultivated potato diversity as a whole.

### 4.2 Ploidy estimation

Comparing diversity at different ploidy levels requires accurate ploidy assignments for informative population genomic analysis. However, the passport data for some individuals had an indicated ploidy that differed from known ploidy for the reported species. For instance, *S. juzepczukii* is generally reported to be triploid (Schmiediche et al., 1982; Spooner et al., 2014; Machida-Hirano, 2015; Graebner et al., 2019; Kyriakidou et al., 2020) but the passport data for *S. juzepczukii* included in this data set listed the accessions as diploid or tetraploid. Passport data is provided by the accession donor and often further characterization data, such as ploidy, is added at the genebank. Ploidy is generally determined by root tip squashes at the genebank (Ordoñez et al., 2017). Accumulation of errors in passport data overtime has been reported in other potato collections (Ellis et al., 2018) and incorrect information regarding ploidy or species can render diversity analysis uninformative. Therefore, we confirmed passport ploidy information using genotype data, based on heterozygosity and allelic frequencies (Gompert and Mock, 2017; Ellis et al., 2018). We found discrepancies in approximately 10% of the accessions and removed them from further analysis. Whether this is the result of inaccurate results from the ploidy calling algorithms, mix-ups within the collection, or variable ploidy within species is unclear.

### 4.3 Diversity in diploids and tetraploids

Cultivated US and European potato is highly heterozygous with on average three haplotypes per locus (Hoopes et al., 2022). Previous studies have suggested that cultivated US tetraploid potato is dramatically more heterozygous than comparable crops, diploid cultivated potato, or wild potato (Hardigan et al., 2017). This pattern is the reverse of the expected loss of diversity associated with crop domestication and improvement (Doebley et al., 2006). However, these were comparisons of observed and expected heterozygosity made without corrections for ploidy. Polyploids appear more heterozygous because there are more opportunities to observe alternate alleles in tetraploids than diploids (2 vs. 4) (Meirmans et al., 2018). Gametic heterozygosity, which compares pairs of alleles in an individual and averages over all possible pairs per loci, is a fairer basis for comparison (Meirmans et al., 2018). When this metric is used, we still observe tetraploids with higher heterozygosity than diploids, but the discrepancy is less dramatic. This suggests that it is not just the opportunity to observe more alleles that leads to higher heterozygosity in polyploids, but also the nature of polyploid genetics. For example, reduced efficacy of selection in polyploids leads to inefficient purging of deleterious alleles (Spooner et al., 2014; Monnahan and Brandvain, 2020).

Among the tetraploids, the group *chilotanum* populations were more heterozygous than the group *andigenum* populations. In particular, US, Canadian, and European populations were the most heterozygous. These populations have been bred to increase heterozygosity based on the assumption of a narrow bottleneck out of South America (Hirsch et al., 2013). Northern group *chilotanum* are the only populations that don’t exhibit low level inbreeding. However, group *chilotanum* also showed the highest expected heterozygosity suggesting that the level of heterozygosity cannot be explained by balancing selection alone. This is consistent with previous observations of high heterozygosity and high haplotype numbers in US potatoes (Hardigan et al., 2017; Meng et al., 2022).

### 4.4 Population structure and admixture

The distribution of diversity across populations is determined by the history of isolation, migration, and cross-pollination between these populations. This history influences relative allele frequencies within and between these populations. The degree to which presumed populations are discreet is referred to as population structure and the shared ancestry of an individual from multiple discreet populations is called admixture.

The primary distinction in our population was between wild and cultivated species. Although, F_ST_ values suggested some admixture between *S. bolivense* and cultivated species. This is consistent with previous reports of low levels of introgression from *S. bolivense* into cultivated US and European potato (Hardigan et al., 2017; Hoopes et al., 2022). Our STRUCTURE results suggest this has been unidirectional introgression from *S. bolivense* into the cultivated species rather than reciprocal admixture. However, the *S. bolivense* samples in this study come from a single population and therefore might not represent all *S. bolivense* populations. Furthermore, they may appear artificially distinct from cultivated material due to high within population relatedness. However, the high number of private alleles found even within one population suggests that *S. bolivense* is distinct from cultivated potato. This is consistent with the general observation that wild relatives have a variety of alleles and desirable traits not present in cultivated material (Jansky et al., 2013; Bamberg and del Rio, 2020).

Population structure within cultivated potato is low with F_ST_ values ranging from 0.019 to 0.059 and the first and second PCA explaining 9.963 and 4.598% of the variation, respectively. Within the diploids, *S. phureja* and *S. stenotomum* are highly similar (**Figure 2**). This is consistent with their membership in a single species in the Spooner taxonomy. However, *S. stenotomum* is more highly admixed with the tetraploid group *andigenum* than *S. phureja* (**Figure 5**) and more heterozygous (**Figure 1A**). Previous analysis of the genebank collection at CIP in Lima Peru found that *S. phureja* made up a distinct population (Ellis et al., 2018). This suggests that there is variation within *S. phureja* as a whole and the 20 individuals in this study differed from those at CIP in that they were more closely related to *S. stenotomum*.

The admixture we observe across ploidy levels is consistent with previous observations in wild and cultivated populations (Kardolus, 1998; Hosaka, 2003; Spooner et al., 2007; Gavrilenko et al., 2010; Rodríguez et al., 2010; Spooner et al., 2012; Gavrilenko et al., 2013; Hardigan et al., 2017; Achakkagari et al., 2020; Hoopes et al., 2022). The high frequency of interploidy crossing in section petota is likely facilitated by the prevalence of unreduced gametes (Watanabe and Peloquin, 1989; Watanabe, 2015). While asymmetrical crossing occurs, for example triploid *S. juzepzukii* is the result of crosses between diploid *S. stenotonum* and the tetraploid wild species *S. acaule* (Kardolus, 1998; Gavrilenko et al., 2010; Rodríguez et al., 2010; Gavrilenko et al., 2013), it generally results in sterile individuals of odd ploidy. Unreduced gametes are necessary for heritable introgression. Contemporary breeders have made extensive use of such crosses to introduce novel traits (Ortiz et al., 1994; Capo et al., 2002; Zimnoch-Guzowska and Flis, 2021; Clot et al., 2023, 2024) and it seems likely that this sort of introgression has been a tool used by humans throughout potato’s history (Hoopes et al., 2022; Meng et al., 2022).

Among the tetraploids we observed separation between the two groups, *andigenum* and *chilotanum* (F_ST_ = 0.023 – 0.1, first PCA explaining 9.9% of the variation). The exception to this is that Peruvian group *chilotanum* shows little differentiation from group *andigenum* (F_ST_ = 0.033 – 0.044) and exhibits the largest *andigenum* component of the group *chilotanum* populations in structure analysis. Peruvian populations from both groups show evidence of introgression from the other (**Figure 6**). In contrast while there is little structure between the Bolivian, Argentinian, Uruguayan, and Chilean group *chilotanum* and *andigenum* populations (F_ST_ = 0.027 – 0.109), there is no evidence of introgression in either direction for these populations (**Figure 6**). This is consistent with Southern South American group *andigenum* being the ancestor of group *chilotanum*, resulting in two highly related taxa in the region. Introgression then, is only possible in diverged Northern populations. This, along with the shared (blue) component in the structure analysis, suggests that there is a geographic aspect as well as a species aspect to population structure in tetraploid cultivated potato.

*S. chilotanum* in the US and Europe is characterized by extensive introgression from wild species (Hardigan et al., 2017; Hoopes et al., 2022). While some of this introgression is the result of post 1945 introgression breeding for disease resistance (Vos et al., 2015) much of it is older (Hoopes et al., 2022; Meng et al., 2022). We observed evidence of introgression into US and European group *chilotanum* from group *andigenum* (**Figure 6**). In general, group *chilotanum* but not group *andigenum* seems to be characterized by continual introgression. The only exception this is in the region where *chilotanum* originated and therefore is least differentiated from *andigenum*. Our observations are consistent with the hypothesis that there has long been extensive mixing in section Petota in Southern South America which is reflected in the genomes of commercial US potatoes.

### 4.5 Core subsets for maintenance of genetic diversity and research cost reduction

Genebanks serve a variety of essential functions including providing a genepool of crop and crop wild relative alleles useful for breeding for novel traits, in particular biotic and abiotic stress resistance. For instance, the US potato genebank preserves germplasm expressing alleles for resistance to soft rot (Ma et al., 2022) and late blight (Enciso-Rodriguez et al., 2018), cold hardiness (Bamberg and Lombard, 2022), freezing tolerance (Bamberg et al., 2020) and increased folate content (Robinson et al., 2019). However, the full extent of potential beneficial alleles within the genebank have not been uncovered and cannot be uncovered unless more screening and evaluation is promoted. In addition, new environmental changes are creating selection pressure at natural habitats which can render new alleles with adaptation and resilience to pests, diseases, and abiotic stresses.

Screening germplasm within a genebank is a large undertaking, which can be made simpler if core collections are available (Frankel, 1984; Gu et al, 2023). Core collections allow breeders and researchers to screen genetically representative germplasm i.e, by maximizing the number of alleles in the minimum number of individuals. For example, in this study all alleles detected in the 730 individuals genotyped can be found in just 329 individuals, dramatically reducing costs and labor involved in screening. For a more realistic number of experimental populations, we found that 77% of the total alleles were captured using just 20 individuals. In the supplement, we provide the recommended subsets generated here (**Supplementary Table S8**, **S9**).

We compared two methods for selecting the accessions to build the core subsets, which produced similar but not identical results, particularly for smaller subsets. All subsets were primarily composed of tetraploid accessions, which probably reflected the higher overall allelic diversity in the tetraploids. An interesting contrast was that GenoCore resulted in a much larger proportion of diploids than the core subset generated by CoreHunter3. In both methods it was observed that the percentage of andigenum germplasm included in the core subsets increased as the subset size became larger.

These subsets provide opportunities for evaluation and screening as they align to previously described core subsets for *S. jamesii*, *S. fendleri*, *S. microdontum*, *S. demissum*, and *S. phureja* available through the US potato genebank (Bamberg and del Rio, 2014; Bamberg et al., 2016; del Rio and Bamberg, 2020, 2021). Unlike some of the previously described core subsets, the ones described here have not been extensively phenotyped. However, they encompass a wider variety of taxa which unlock opportunities for screening and studying traits of interest. The individual accessions genotyped in this study are available through GRIN.

## Data availability statement

The datasets presented in this study can be found in online repositories. The names of the repository/repositories and accession number(s) can be found below: PRJNA1111837 (SRA). The vcf files and scripts used for analysis are available at https://github.com/shannonlabumn/.

## Author contributions

HT: Writing – review & editing, Writing – original draft, Investigation, Formal analysis, Data curation, Conceptualization. ADR: Writing – review & editing, Resources, Funding acquisition, Data curation, Conceptualization. JB: Writing – review & editing, Resources, Funding acquisition, Data curation, Conceptualization. LS: Writing – review & editing, Writing – original draft, Supervision, Funding acquisition, Conceptualization.
