# Supplementary figures and images for: Potato soup: analysis of cultivated potato gene bank populations reveals high diversity and little structure
Source: Front Plant Sci. 2024 Jul 18;15:1429279. doi: 10.3389/fpls.2024.1429279 (PMC11291250; doi:10.3389/fpls.2024.1429279)

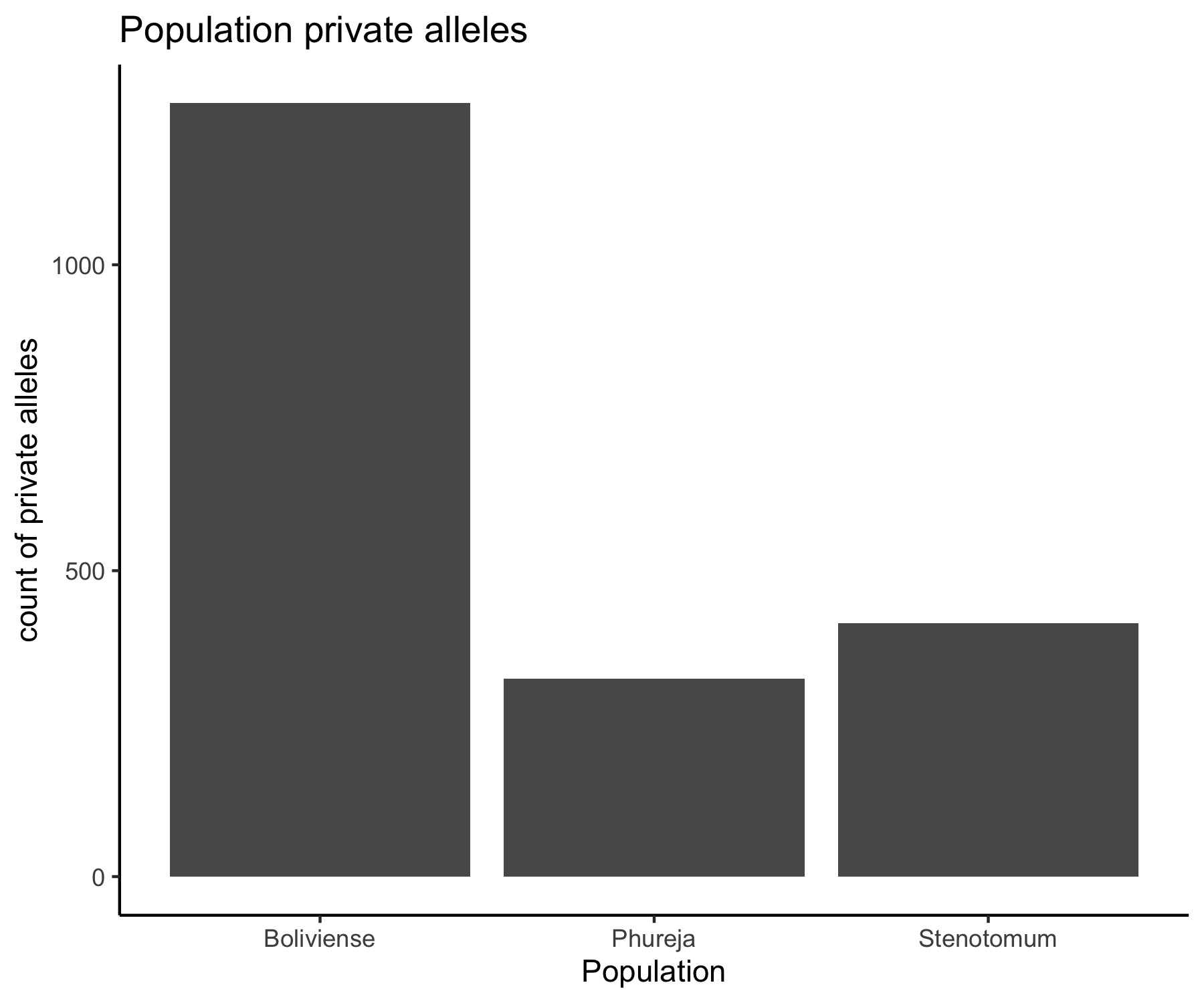

Supplement: Supplementary file 1 [file DataSheet_1.zip › SUPPLEMENTAL/Supplemental_figure_2_private_alleles.jpg]

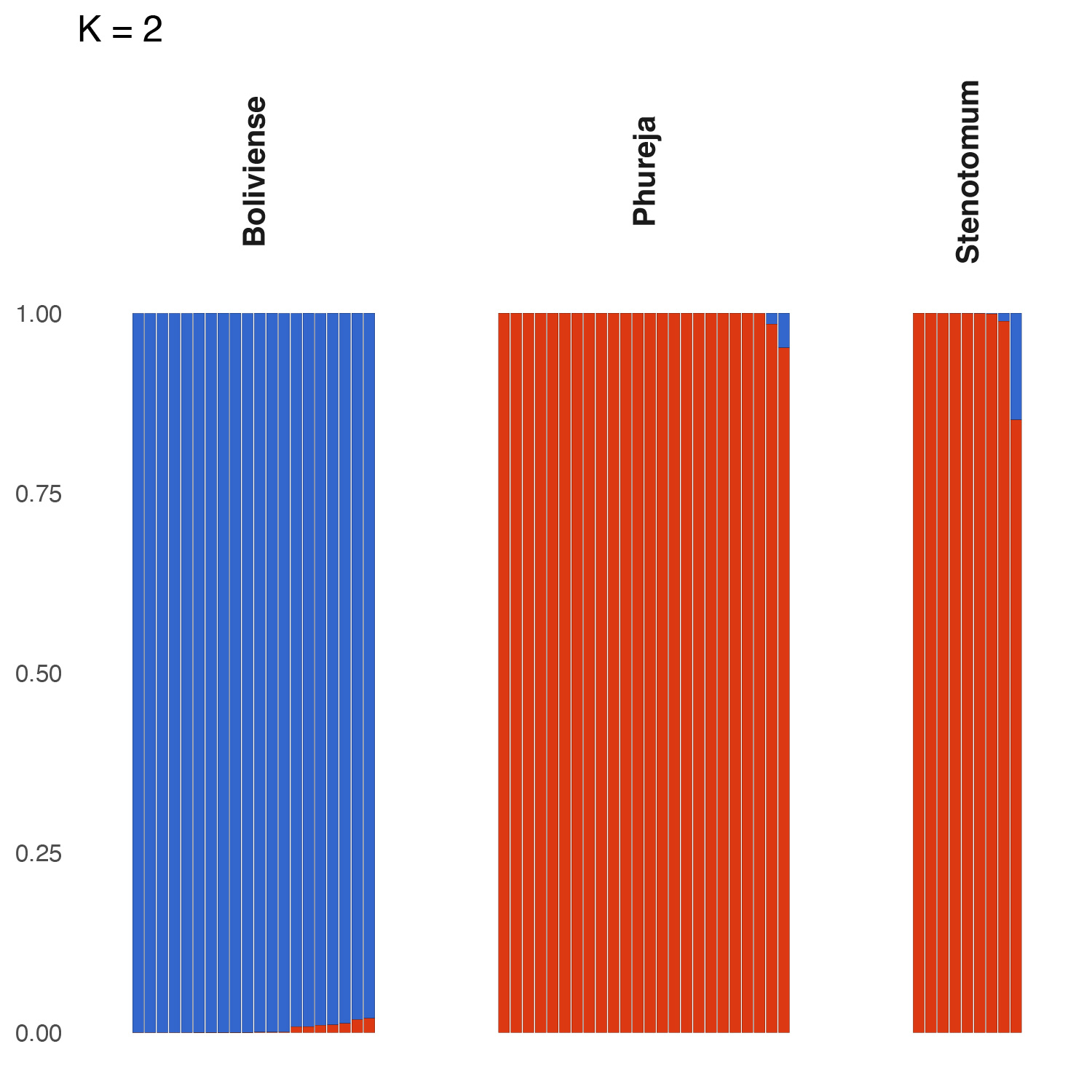

Supplement: Supplementary file 1 [file DataSheet_1.zip › SUPPLEMENTAL/Supplemental_Figure_4_Tuttle_etal_1223.jpg]

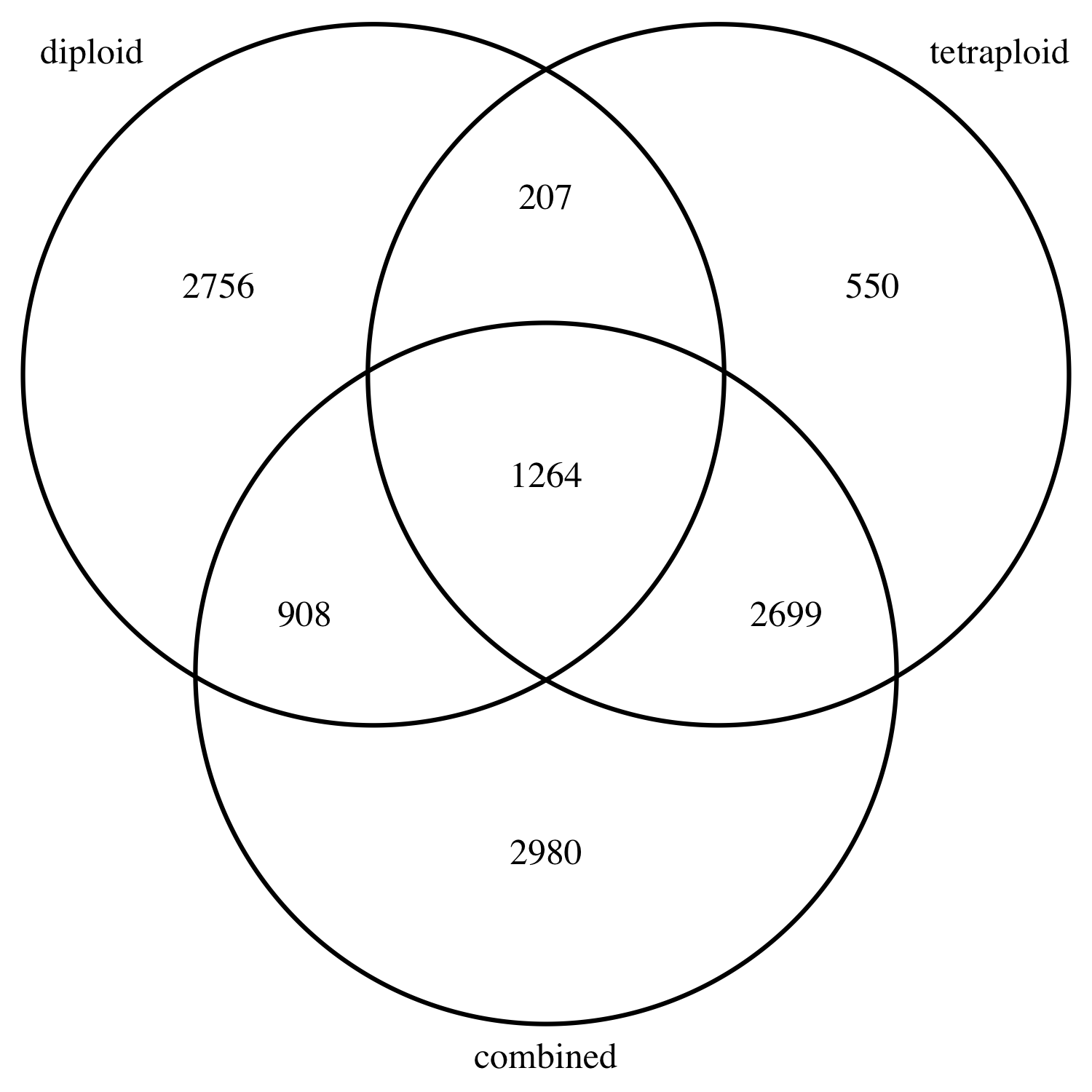

Supplement: Supplementary file 1 [file DataSheet_1.zip › SUPPLEMENTAL/Supplemental_Figure_1_overlapTuttle.png]

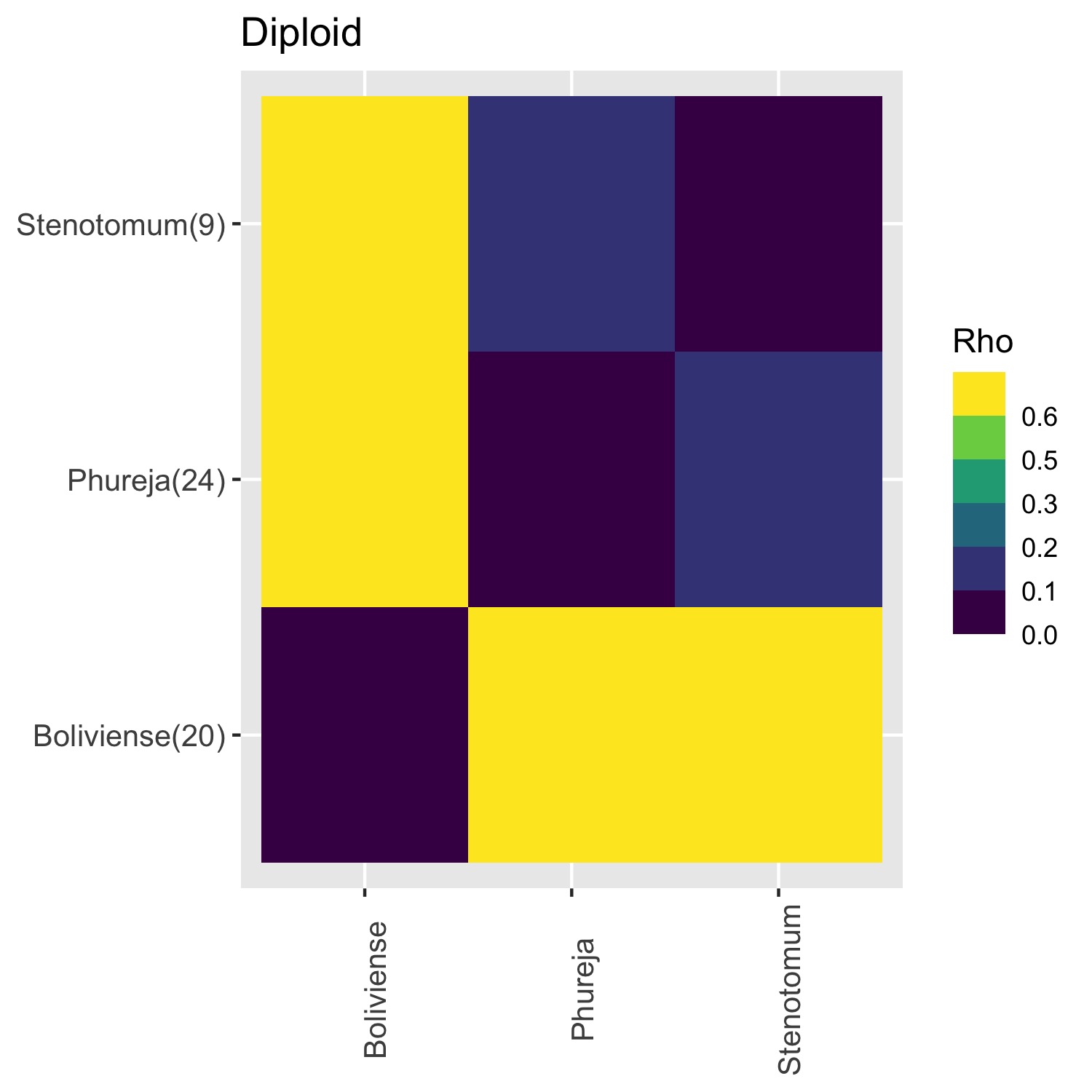

Supplement: Supplementary file 1 [file DataSheet_1.zip › SUPPLEMENTAL/Supplemental_Figure_3_rho.jpg]

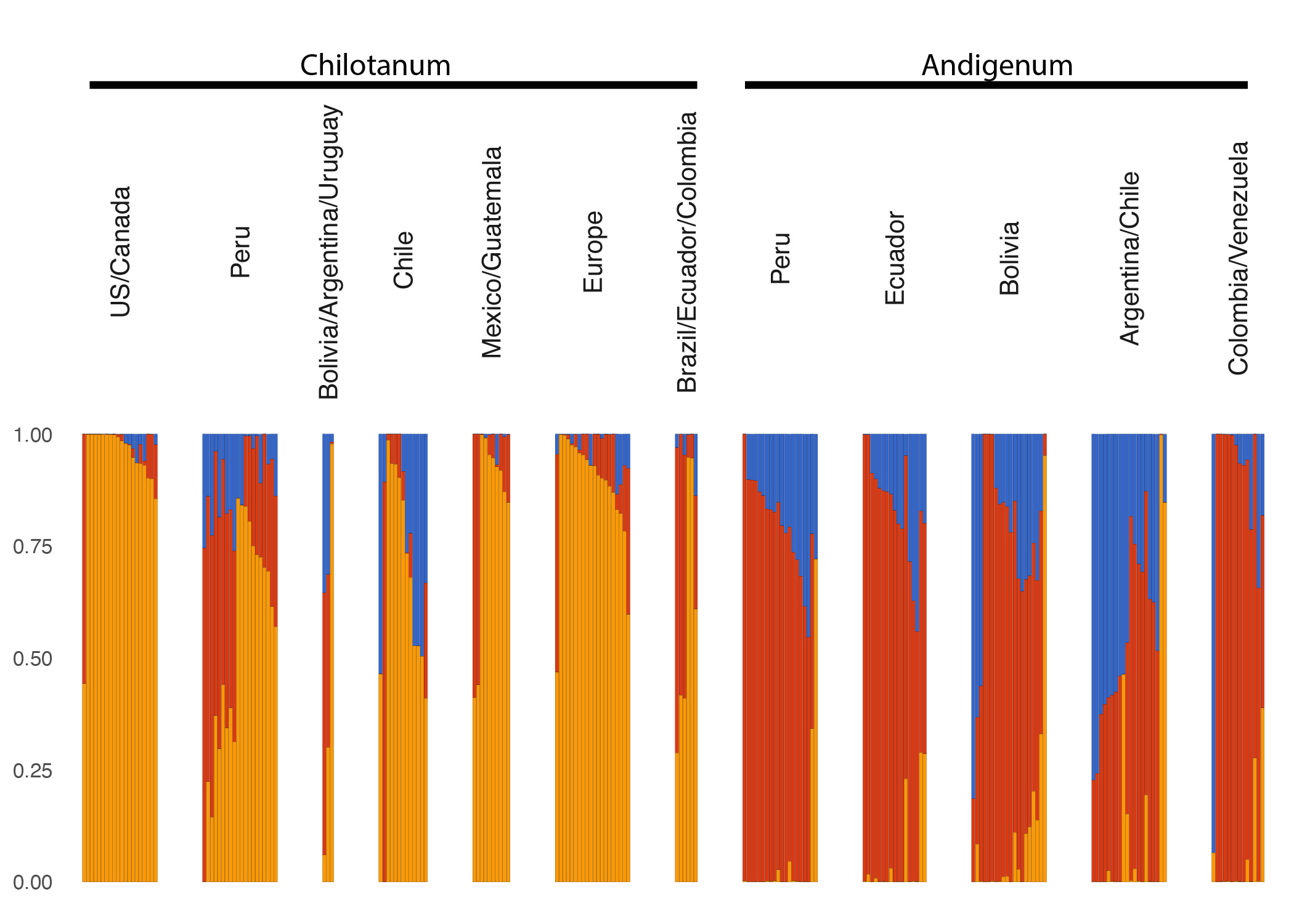

Supplement: Supplementary file 1 [file DataSheet_1.zip › SUPPLEMENTAL/Suplemental_Figure_5_Tuttle.jpg]
